# Female Sex and Living in a Large City Moderate the Relationships between Nursing Students’ Stress Level, Perception of Their Studies, and Intention to Practice Professionally: A Cross-Sectional Study

**DOI:** 10.3390/ijerph19095740

**Published:** 2022-05-09

**Authors:** Natalia Dominika Pawlak, Lena Serafin, Bożena Czarkowska-Pączek

**Affiliations:** 1 Department of Clinical Nursing, Health Sciences Faculty, Medical University of Warsaw, Erazma Ciolka Street 27, 01-445 Warsaw, Poland; lena.serafin@wum.edu.pl (L.S.); bozena.czarkowska-paczek@wum.edu.pl (B.C.-P.); 2 Doctoral School, Medical University of Warsaw, Zwirki i Wigury Street 61, 02-091 Warsaw, Poland

**Keywords:** nursing student, stress, professional practice

## Abstract

One way to increase nursing retention is to expand the number of nursing education programs; however, a more cost-effective initial step would be to ensure that each graduate will start a professional career. Nursing studies expose students to prolonged and uncontrolled stress that negatively affects their professional identity and health. Two hundred and fifty-four nursing students participated in this study. The data were obtained using the Perceived Stress Scale (PSS-10), a proprietary questionnaire on the students’ perception of their study, intention to practice in the future, and other metrics. Among our sample, a dozen students were unsure that they would enter the nursing profession. Stress levels in women were higher than in men. Respondents indicated that they were afraid of the return of the pandemic. This analysis was significant among people living in large cities. Based on our findings, five themes should be prioritised: favourable study environment and adequate competencies (including implementation of stress management techniques, especially among women and students living and studying in large cities), appropriate working hours, quality of practical classes, and quality of personal protective equipment.

## 1. Introduction

The shortage of nurses that results from low nurse retention, defined as “keeping nurses in their jobs”, and instability in the nursing workforce are major global concerns [1,2]. The number of nurses per 1000 inhabitants varies widely among countries. Of those countries reporting to OECD, besides the Slovak Republic, this number decreased in 2020 compared to 2019. The mean number of nurses per 1000 inhabitants in OECD countries in 2019 was 8.83, while, in 2020, this decreased to 6.98 [3,4]. Moreover, there is evidence that more nurses retire than enter the profession every year, contributing to the nurses’ constantly increasing mean age and lowering nurse retention [5]. The World Health Organization estimates that the global needs-based shortage of nurses and midwives will be greater than 9 million by 2030 [6]. There are several ways to counteract the nursing shortage, such as pull and push factors, including appropriate and supporting working conditions, limiting working pressures, appropriate wages, career development and opportunities, and improving the social image of the profession [7]. Then, these factors are also crucial to maintaining healthcare workers’ mental health and their ability to deliver effective patient care [8,9]. Another way to increase nursing retention is to expand the number of nursing education programs and ensure that each graduate will start a professional career. This also counteracts the ageing of the nursing population.

One way to increase the number of nursing students and graduates is to ensure a supportive learning environment and appropriate acquisition of knowledge and practical skills resulting in the readiness to professionally practice. According to the Directive 2005/36/EC, and later 2013/55/EU [10], basic nursing education comprises 4600 h of training, half of which is practical and at least one-third is theoretical. The nursing educational program obliges the nursing student to plan, organise, evaluate, and communicate the nursing care given directly with a healthy or sick individual [10]. In many countries, including Poland, nursing education programs lead to the obtaining of a practice license that is valid for 3 years, which means about 1500 education hours per year, making the program especially challenging. Thus, nursing studies expose students to prolonged and uncontrolled stress that negatively affects their professional identity and health [11].

Therefore, this study was conducted to determine the impact of stress levels on those intending to pursue professional practice among last-year nursing students and to determine the impact of sex, place of residence, and type of university on the relationship between their stress level, perception of the study, and intention to professionally practice in the future.

## 2. Materials and Methods

### 2.1. Design and Sample

This was a correlational cross-sectional study of the bachelor’s degree nursing students in their final year. The study was performed from January to April 2021. The inclusion criteria were being in the last year of undergraduate studies in the field of nursing and not practicing nursing or other medical professions. Convenience sampling was used in this study and included 254 participants. The minimum required sample size was calculated *a priori* using G*Power software, assuming a confidence interval of 95%, a statistical power of 80%, and an α of 0.05. The minimum sample size was 186, and 260 students completed the questionnaire. After data analysis, six participants were excluded because they did not meet the inclusion criteria.

### 2.2. Instrument

This work used an online survey using Google Forms. The invitation to participate was shared via social media groups focused on nursing students.

Data were obtained using two questionaries. The first was the Polish adaptation of Perceived Stress Scale (PSS-10). The second was developed in house and included 5 questions regarding their attitude to nursing studies and plans for future professional life. Because the COVID-19 pandemic increased the awareness of difficulties and threads connecting with nursing practice, we also asked the responders how the pandemic has impacted their professional choices. An additional part of the questionnaire was a metric that allowed us to characterize the study group.

The PSS-10 was developed by Cohen et al. [12], and its Polish validity and reliability study was conducted by Juczyński and Ogińska-Bulik [13]. It uses a five-point Likert-type scoring system. Each item on the scale is scored with options such as “never = 0 points”, “almost never = 1 point”, “sometimes = 2 points”, “fairly often = 3 points”, and “very often = 4 points”. The overall score of the scale is the sum of all points with a theoretical distribution from 0 to 40. A higher score implies a greater level of perceived stress. The internal reliability coefficient of the items regarding perceived stress on the Polish version of scale is 0.86 [13]. In our study, the Cronbach’s α coefficient for the PSS-10 scale was 0.714.

The second part of the questionnaire consisted of 5 questions about the perception of the nursing studies and intent to pursue professional practice after completing the study program. The 5-point Likert scale from 0 “never” to 4 “very often” was again used to answer the statements. These questions have been developed by authors based on a literature review [11,14,15,16]. Sociodemographic data collection was performed at the end of the questionnaire. The questions concern age, sex, type of university, year of study, and place of residence.

### 2.3. Ethical Consideration

This study was approved by the Medical University of Warsaw Review Board (AKBE/2/2021). Before answering, the students were informed about the aim of the study, inclusion criteria, the voluntary and anonymous nature of participation, and the right to withdraw from the study with no specific explanation needed. On the first page of the online link, students were asked to check the statement agreeing to participate with an understanding of the research assumptions. The survey would not open until the box was checked. Participants received no compensation for completing the survey.

### 2.4. Data Analysis

Statistical analyses used IBM SPSS Statistics 25.0 (Predictive Solutions, Cracow, Poland). Spearman correlation was performed to establish the relationship between the variables. A moderation analysis was performed using A. Hayes’ macro-PROCESS to establish the moderating role of sociodemographic variables for the relationship between answers to inhouse questions and stress (2017). A linear regression analysis was performed using the stepwise method to determine whether the sociodemographic variables were predictors of the stress level. The level of significance was α = 0.05.

## 3. Results

### 3.1. Students’ Characteristics

The study included 254 nursing students in their final year. Table 1 presents the socio-demographic characteristics of the study group (sex, age, universities, place of residence). Place of residence has been presented based on the stratification of cities according to the Central Statistical Office in Poland (small cities, medium-sized cities, large cities, and the largest cities in Poland) [17]. The mean age of the participants was 22.8 years. The youngest person was 20 and the oldest was 47 years old.

### 3.2. Students’ Stress Level

The mean PSS-10 score was 24.95 (SD = 5.64). The minimum value in this scale was 0 points, and the maximum was 40 points. The analysis showed that the distribution of results is left-skewed, which indicates that most of the respondents obtained results above the group mean, suggesting a higher level of stress. Linear regression analysis was performed using the stepwise method to determine whether sex, place of residence, and type of university were significant predictors of the stress level. This step only used those predictors that met the probability criteria F < 0.05. The model was well suited to the data: F (1.252) = 7.60; *p* = 0.006. The analysis showed that sex was the only significant predictor of the perceived stress level (*B* = −3.07; *SE* = 1.11; *p* = 0.006). Stress levels in women were on average 3.07 points higher than in men. Sex accounted for 2.9% of the stress level variance.

### 3.3. Students’ Perception of Their Study and Future Practice

Many of respondents (37.8%; *n* = 97) presented, at least sometimes, a feeling of regret in choosing nursing as a field of study. Moreover, many students had not finally decided to work in the profession after graduation (78%; *n* = 198). Most respondents frequently thought that distance learning and the limitations of practical classes had a negative impact on their practical skills (73.1%; *n* = 185). As many as 80.8% (*n* = 204) of respondents indicated that they very often or fairly often were aware that the pandemic could repeat itself. Detailed results regarding the students’ perception of the study and their intention to professional practice are presented in Table 2. 

### 3.4. The Relationship between Stress Levels and Students’ Perception of Their Study and Intention to Professional Practice

There was no correlation between stress and the willingness to work in the profession (r = 0.10, *p* = 0.110). There was also no correlation between the level of stress and limited practical activities at the bedside on practical skills (r = 0.11, *p* = 0.069). However, the analysis showed the relationship between stress level and concern about the lack of personal protective equipment in the facility (r = 0.28, *p* < 0.001) and awareness that the current pandemic situation related to COVID-19 may repeat itself (r = 0.20. *p* = 0.001).

### 3.5. Moderators for the Relationship between Stress Levels and Students’ Perception of Their Study and Intention to Professional Practice

A moderation analysis was performed using A. Hayes’ PROCESS to determine whether sex, type of university, and place of residence is a moderator of the relationship between responses to questions related to the students’ perception of their study and intention to professionally practice. 

In relation to sex, of the five analyzed models, only one showed a significant moderation effect—sex moderated the relationship between regretting the choice of studying nursing and stress. The model fit the data well; F (3.250) = 6.50; *p* < 0.001, which explains the 7.2% of the dependent variable variance. Table 3 presents the regression coefficients of the analyzed model.

A detailed analysis of simple effects showed that there was no significant relationship between regret choosing a field of study and stress among women (*B* = 0.50; *SE* = 0.31; *p* = 0.106); this was significant in men (*B* = 2.32; *SE* = 0.77; *p* = 0.003; *95%CI* [0.79; 3.84]). Stress was higher when they regretted choosing a field of study. These results are illustrated in Supplementary Figure S1.

The analysis of the moderation of five models in which the type of university was included as the moderator did not show a significant moderating role of the type of university. Therefore, these models will not be described. An analysis of the moderation of five models in which the place of residence was included as a moderator showed a moderating role of two models; these are discussed below.

The first model considered the moderating role of the place of residence for the relationship between the answer to the question “Do you think to starting working in the profession after graduation?” as well as perceived stress. The model turned out to be a good fit for the data (F (9.244) = 2.21; *p* = 0.022) and explained 7.5% of the dependent variable’s variance. The overall (higher order) interaction effect was statistically significant (F (4.244) = 3.12; *p* = 0.016), and its inclusion increased the percentage of explained variance by 4.7%. The regression coefficients for the discussed model are presented in Table 4.

The analysis of simple effects showed that a significant relationship between the decision to work in the profession after graduating from nursing studies and stress was significant only in the case of people living in cities with more than 500,000 residents. A higher willingness to work in the profession after graduation implied a higher stress level. The effects were insignificant in the remaining groups (Table 5; Supplementary Figure S2).

The second model considered the moderating role of the place of residence for the relationship between perceived stress and the answer to the following question: “Are you aware that the current COVID-19 pandemic may repeat itself?” The model turned out to be a good fit for the data (F (9.244) = 3.82; *p* < 0.001) and explained 12.4% of the dependent variable’s variance. The regression coefficients for the model in question are shown in Table 6.

The overall (higher order) interaction effect was statistically significant (F (4.244) = 3.62; *p* = 0.007), and its inclusion increased the percentage of explained variance by 5.2%. The regression coefficients for the discussed model are presented in Table 6.

The analysis of simple effects showed that a significant relationship between the answer to the question regarding awareness of the possibility of repeating the current situation with COVID-19 and stress was significant among people living in cities from 151,000 to 500,000 inhabitants as well as in cities with more than 500,000 residents. More awareness correlated with a higher stress level. The effect was insignificant in smaller towns and among people living in the countryside (Supplementary Figure S3; Table 7).

## 4. Discussion

Taking care of adequate nursing resources begins with ensuring the appropriate and adequate number of nursing students. Another problem is to ensure that every graduate will start their professional career. Among our sample, more than a dozen students were unsure that they would enter the nursing profession. Therefore, it is important to make nursing studies friendly for students and to provide them with the appropriate knowledge, skills, and social competencies that will allow them to start their jobs with the feeling that they are properly prepared. Supporting future healthcare professionals, both pre- and post-graduation, seems crucial in solving the global nursing shortage [16].

Additionally, the COVID-19 pandemic has seriously impacted the learning conditions and has demonstrated that the profession of a nurse is particularly difficult, dangerous, possibly health- and life-threatening, and has revealed in the majority of health care facilities a lack of preparation in terms of ensuring epidemiologically safe conditions for medical personnel, including nurses, which results in high levels of stress and anxiety [18]. Thus, this possibly deepened the students’ fear regarding the future professional environment in terms of physical and psychological threats and therefore impacted the decision to start a nursing career after studies were completed. Yang et al. showed in general that students who experienced or witnessed stressful events related to COVID-19 reported negative psychological symptoms [19]. These symptoms could be manifested by a sense of tension, fear of infection, insomnia, and depressed mood [15,19]. In general, nursing students experience greater stress levels than other college students [20], and this could affect patients’ outcomes, patient safety, and quality of care [21]. Undergraduate nursing students are exposed to stressful situations, especially those in the more clinically-oriented years of training [22]. Our study revealed that nursing students perceived a moderate stress level (24.91 ± 5.65), though most respondents reported higher than all group mean stress levels. In Aslan and Pekince [14], the stress level of Turkish nursing students measured by using the same tool during the pandemic was 31.69 ± 6.91. This level was considered moderate. Nursing students normally presented moderate stress levels [23,24,25,26]. Previous research has shown that stress levels decreased with higher knowledge about the pandemic [27,28]. Nursing students are a very specific group of students and are directly integrated into the pandemic given their health training and area of knowledge [18]; thus, their stress level is not higher than before the pandemic [14]. Erisn [11] showed that health awareness was high [11]. This may be because students receive reliable knowledge in medicine and thus try to translate this information into future professional practice. However, education regarding infection prevention and protection measures should be incorporated into the study program to a greater extent. Especially here, respondents frequently realise a pandemic may reoccur, and specialists are alarmed that future pandemics are inevitable [29].

We did not confirm the correlation between stress level and willingness to work in nursing. Despite the fact that a large percentage of the respondents considered that the limitation of practical classes influences the development of their skills, we did not confirm the relationship between stress level and limited practical classes in real contact with the healthy or sick individual, which is essential for providing appropriate professional skills among graduates. Organising such classes for nursing students is associated with several difficulties, such as accelerated patient processing, in which patients are discharged from hospitals earlier, reductions in the number of hospital beds on the wards where students mainly have their clinical practice, or limited space in highly specialised wards that can only accommodate a limited number of students. Therefore, additional solutions should be implemented to provide an appropriate number of hours of practical training, for instance, partial training in simulation centres. This takes additional significance in the context of a potential reoccurrence of pandemic and the necessity to implement e-learning and distance learning [30] to limit the spread of SARS-CoV-2 or another microorganism [31]. The effectiveness of simulation learning has been shown in different nursing areas to be as good as or even better than traditional learning; however, the EU requirements describe clinical learning as being ‘in direct contact with a healthy or sick individual’ [32]. Thus, the revision of these requirements should be alternatively considered. Interestingly, the students’ sociodemographic characteristics are related to their stress level [14,33]. Here, only sex was a significant predictor of perceived stress. Even though men in our study accounted for only 11.4% of respondents, this is still a lot compared to the percentage of men in nursing in Poland, which is 2.7% [34]. Female students showed higher rates of perceived stress than men. This result agrees with previous studies [14,35,36,37]. Long-term analysis revealed that women themselves attach more importance to their inner experiences: they are more vulnerable to depression, anxiety, and loneliness [38]. We found that only men who said they regretted choosing a field of study had higher stress. This means that stress in men causes an active attitude towards a potential stressor that may make it easier to cope, which is worth considering in future research. Therefore, supporting nursing students, most of whom are female, in managing stress is an important aspect of preparing them for professional nursing practice. It is important to support nursing students in coping with stress, thus reducing, for instance, the risk of premature termination of professional education. Often, instructors do not realise the causes or extent of students’ stress; however, a deliberate engagement with students about their stress and offering interventions can be beneficial [39]. Therefore, it is critical for the faculty to monitor students’ stress and provide opportunities for frequent debriefing sessions, particularly during times of crisis such as a pandemic [15].

There was a correlation between nursing students’ residence and their perceived stress level. Nursing students who declared that they lived in larger cities during their study or stayed at the place of study far from their relatives might have had more stress. Üstün found that the anxiety levels of participants who were away from their families and social life and those who felt lonely were significantly higher than other participants [40]. A pandemic could impact this correlation. Living in larger cities with restrictions around social distancing can be more stressful. Bai et al. found that nursing students living in rural areas during the pandemic were more likely to choose nursing as their future career than their counterparts living in urban areas [41].

We found that the strongest relationship was between stress and concern about the facility’s lack of personal protective equipment. Studies had shown that students had increased fear and anxiety in clinical settings when personal protective equipment was deficient [23,42]. According to Savitsky et al., 50% of students working in healthcare settings lacked personal protective equipment at work; moreover, among these, the level of anxiety was higher. This anxiety continued into the current academic year and increased [23]. Preventive measures provide better comfort at work, reduce anxiety levels, and negate disease risk.

## 5. Limitations

This study has some limitations. First, the cross-sectional design cannot establish a causal link between the investigated variables and does not allow us to formulate conclusions regarding the long-term effects of stress. Therefore, a longitudinal design is recommended for future studies. Second, convenience sampling does not generalise the results among nursing students. A randomised sample from different nursing schools is suggested to further study this issue.

## 6. Conclusions

Adequate nursing retention relies on many factors, including the number of nursing students. The development and monitoring of the intention to work in the nursing profession should be constantly implemented by the education environment. Monitoring stress levels among nursing students and supporting career decisions should be performed in centres of nursing education considering the nursing shortage. An appropriate and favourable study environment and adequate competences should be provided to nursing students. These include the implementation of stress management techniques, especially among women and students living and studying in large cities, an appropriate number of hours and quality of practical classes providing adequate competences and skills, and personal protective equipment in the healthcare facility. Due to the common awareness that a reoccurrence of the COVID-19 pandemic is possible, knowledge regarding the pandemic and alternative ways of conducting practical classes should be considered.

## Figures and Tables

**Table 1:** Students’ characteristics (N = 254).

Characteristics of Respondents		N	%
Sex	Women	225	88.6
	Men	29	11.4
Age	≤20–25	231	90.9
	26–30	10	3.9
	31–25	6	2.4
	>36	7	2.8
University	Medical	241	94.9
	Vocational	13	5.1
Place of residence	Village	84	33.1
	City up to 50,000 residents	26	10.2
	City from 50,001 to 150,000 residents	30	11.8
	City from 150,001 to 500,000 residents	20	7.9
	City > 500,000 residents	94	37

**Table 2:** Students’ perception of their study and intention to professional practice (N = 254).

	Never		Hardly Ever		Sometimes		Quite Often		Very Often	
	N	%	N	%	N	%	N	%	N	%
Do you regret choosing a field of study (nursing)?	**115**	**45.3**	41	16.1	67	26.0	19	7.5	12	4.7
Do you think that limiting practical work at the bedside may adversely affect the development of your practical skills?	15	5.9	16	6.3	38	15.0	**41**	**16.1**	**144**	**57.0**
Do you think to starting working in the profession after graduation?	7	2.8	8	3.1	41	16.0	**56**	**22.0**	**142**	**56.0**
Are you worried about the lack of personal protective equipment at the facility where you decide to work?	37	15.0	24	9.4	64	25.0	51	20.1	78	31.0
Are you aware that the current COVID-19 pandemic may repeat itself?	5	2.0	4	1.6	41	16.0	**53**	**20.8**	**151**	**60.0**

**Table 3:** Regression coefficients for the model considering the moderating role of sex for the relationship between regret choosing a field of study and stress.

					*95% CI*	
	*B*	*SE*	*t*	*p*	*LL*	*UL*
Constant	24.91	0.34	72.60	<0.001	24.24	25.59
Do you regret choosing a field of study (Nursing)?	0.70	0.29	2.44	0.015	0.14	1.26
Sex	−3.51	1.10	−3.18	0.002	−5.68	−1.34
Interaction	1.82	0.83	2.18	0.030	0.17	3.46

*B*—unstandardized regression coefficient; *SE*—standard error; *t*—t-statistic; *p*—*p*-value; *CI*—confidence interval; *LL*—lower level; *UL*—upper level.

**Table 4:** Regression coefficients for the model considering the moderating role of the place of residence for the relationship between the decision to go to work after graduation from nursing and stress.

					*95% CI*	
	*B*	*SE*	*t*	*p*	*LL*	*UL*
Constant	25.32	0.60	42.05	<0.001	24.14	26.51
Do you think to starting working in the profession after graduation?	−0.67	0.66	−1.02	0.310	−1.98	0.63
W1	−1.04	1.24	−0.84	0.402	−3.49	1.40
W2	−0.58	1.21	−0.48	0.632	−2.97	1.81
W3	−1.17	1.38	−0.85	0.397	−3.90	1.55
W4	−0.11	0.83	−0.13	0.897	−1.75	1.53
Interaction 1	−0.93	1.46	−0.64	0.525	−3.80	1.94
Interaction 2	1.81	1.25	1.45	0.148	−0.65	4.27
Interaction 3	2.19	1.12	1.96	0.051	−0.01	4.39
Interaction 4	2.42	0.84	2.88	0.004	0.77	4.07

Annotation. Interaction 1: question × W1; Interaction 2: question × W2; Interaction 3: question × W3; Interaction 4: question × W4. *B*—unstandardized regression coefficient; *SE*—standard error; *t*—t-statistic; *p*—*p*-value; *CI*—confidence interval; *LL*—lower level; *UL*—upper level.

**Table 5:** Simple effects coefficients for the place of residence in the context of the relationship between the decision to work after graduation in nursing and stress.

	*B*	*SE*	*p*	*95% CI*
Village	−0.67	0.66	0.310	−1.98; 0.63
City up to 50,000 residents	−1.60	1.39	0.219	−4.16; 0.96
City from 50,001 to 150,000 residents	1.14	1.06	0.283	−0.95; 3.22
City from 150,001 to 500,000 residents	1.51	0.90	0.093	−0.25; 3.28
City > 500,000 residents	1.75	0.52	0.001	0.73; 2.76

*B*—unstandardized regression coefficient; *SE*—standard error; *p*—*p*-value, *CI*—confidence interval.

**Table 6:** Regression coefficients for the model considering the moderating role of the place of residence for the relationship between awareness of the possibility of a repetition of the current pandemic situation and stress.

					*95% CI*	
	*B*	*SE*	*t*	*p*	*LL*	*UL*
Constant	25.25	0.58	43.24	<0.001	24.10	26.40
Are you aware that the current COVID-19 pandemic may repeat itself?	0.25	0.65	0.38	0.706	−1.04	1.53
W1	−0.80	1.21	−0.66	0.511	−3.19	1.59
W2	−0.16	1.16	−0.14	0.889	−2.45	2.12
W3	−0.61	1.34	−0.45	0.651	−3.26	2.04
W4	−0.19	0.81	−0.23	0.818	−1.78	1.40
Interaction 1	−0.72	1.23	−0.59	0.557	−3.15	1.70
Interaction 2	0.74	1.25	0.59	0.554	−1.71	3.21
Interaction 3	2.87	1.13	2.54	0.012	0.65	5.10
Interaction 4	2.45	0.89	2.75	0.006	0.69	4.20

Annotation. Interaction 1: question × W1; Interaction 2: question × W2; Interaction 3: question × W3; Interaction 4: question × W4. *B*—unstandardized regression coefficient; *SE*—standard error; *t*—t-statistic; *p*—*p*-value; *CI*—confidence interval; *LL*—lower level; *UL*—upper level.

**Table 7:** Simple effects coefficients for the place of residence in the context of the relationship between awareness of the possibility of a repetition of a pandemic situation and stress.

	*B*	*SE*	*p*	*95% CI*
Village	0.24	0.65	0.706	−1.04; 1.53
city up to 50,000 residents	−0.48	1.04	0.649	−2.53; 1.58
city from 50,001 to 150,000 residents	0.99	1.07	0.356	−1.12; 3.09
city from 150,001 to 500,000 residents	3.12	0.92	0.001	1.30; 4.93
city > 500,000 residents	2.69	0.60	<0.001	1.50; 3.88

*B*—unstandardized regression coefficient; *SE*—standard error; *p*—*p*-value, *CI*—confidence interval.

## Data Availability

The datasets used and analyzed during the current study are available from the corresponding author upon reasonable request.

## Supplementary Materials

The following supporting information can be downloaded at: https://www.mdpi.com/article/10.3390/ijerph19095740/s1, Figure S1: Relationship between regretting choice of major and stress level with considering sex.; Figure S2: The relationship between the decision to pursue a career after graduation and stress levels by place of residence.; Figure S3: The relationship between awareness of the possibility of a repeat of the current pandemic situation and stress levels by place of residence.
