# Supplementary material for: Female Sex and Living in a Large City Moderate the Relationships between Nursing Students’ Stress Level, Perception of Their Studies, and Intention to Practice Professionally: A Cross-Sectional Study
Source: Int J Environ Res Public Health. 2022 May 9;19(9):5740. doi: 10.3390/ijerph19095740 (PMC9099526; doi:10.3390/ijerph19095740)
Supplement: Supplementary file 1 [file ijerph-19-05740-s001.zip › ijerph-1666238-supplementary.pdf]

Supplementary Materials

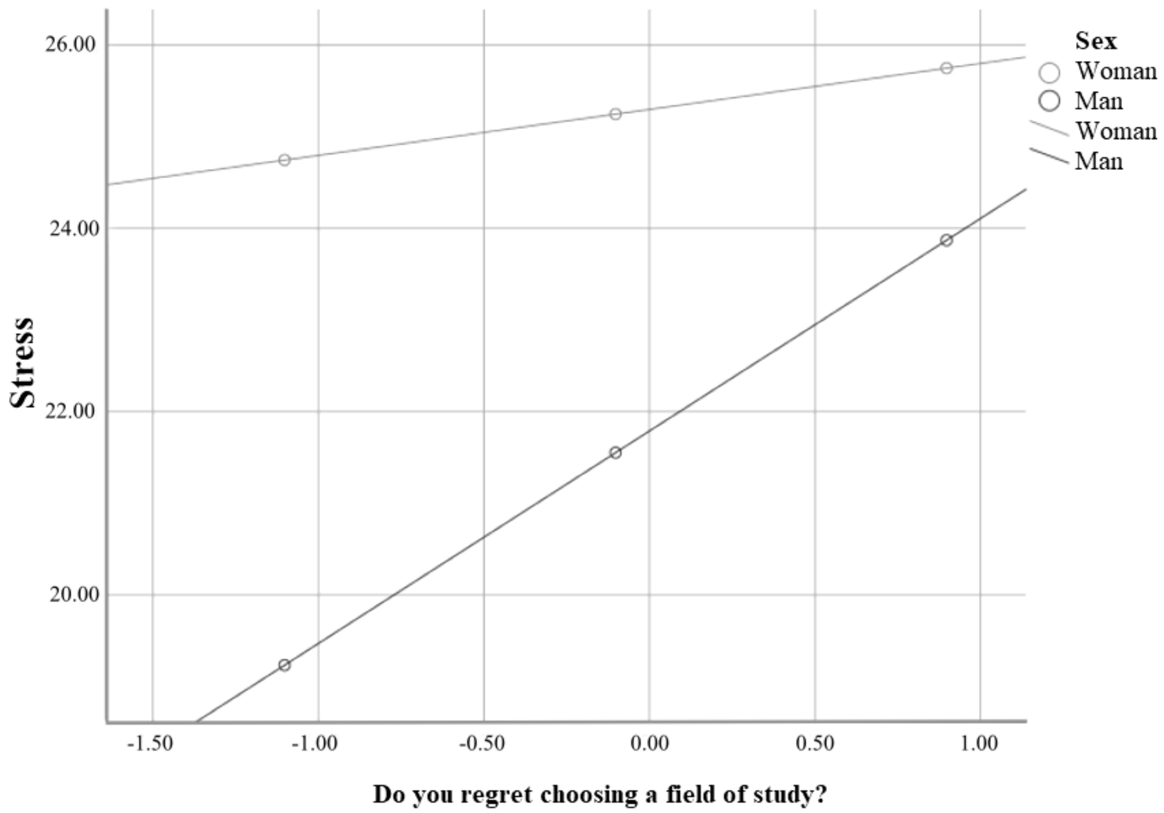

Figure S1. Relationship between regretting choice of major and stress level with considering sex.

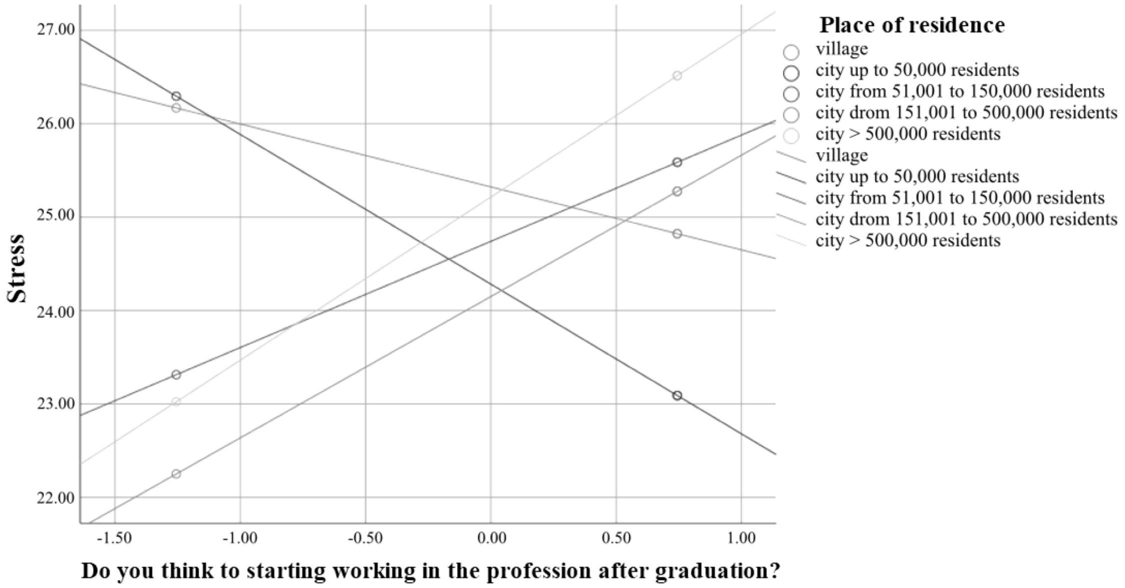

Figure S2. The relationship between the decision to pursue a career after graduation and stress levels by place of residence.

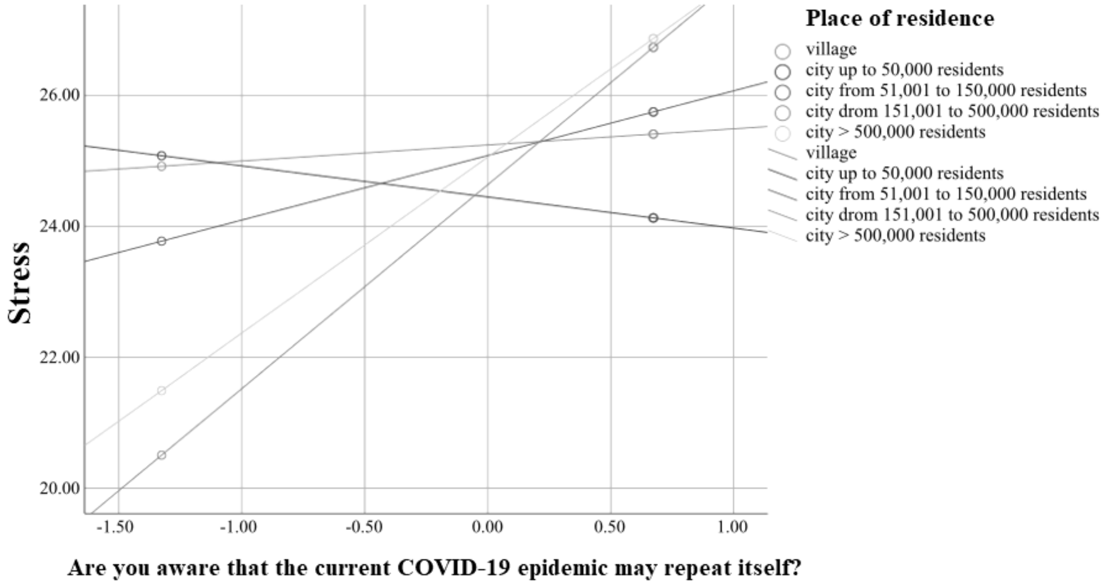

Figure S3. The relationship between awareness of the possibility of a repeat of the current pandemic situation and stress levels by place of residence.
